# Supplementary material for: Biomimetic nanovaccine-mediated multivalent IL-15 self-transpresentation (MIST) for potent and safe cancer immunotherapy
Source: Nat Commun. 2023 Oct 24;14:6748. doi: 10.1038/s41467-023-42155-z (PMC10598200; doi:10.1038/s41467-023-42155-z)
Supplement: Supplementary file 1 — Supplementary Information [file 41467_2023_42155_MOESM1_ESM.pdf]

Supplementary Information

Biomimetic nanovaccine-mediated multivalent IL-15 self-transpresentation (MIST) for potent and safe cancer immunotherapy

Author list: Kaiyuan Wang[1,2#], Xuanbo Zhang[1,2#], Hao Ye[1,3#], Xia Wang[4], Zhijin Fan[5], Qi Lu[1], Songhao Li[1], Jian Zhao[1], Shunzhe Zheng[1], Zhonggui He[1*], Qianqian Ni[2,6,7*], Xiaoyuan Chen[2,6,7,8*], Jin Sun[1*]

Affiliations:

[1]Department of Pharmaceutics, Wuya College of Innovation, Shenyang Pharmaceutical University, 103 Wenhua Road, Shenyang Liaoning, 110016, P. R. China

[2]Departments of Diagnostic Radiology, Surgery, Chemical and Biomolecular Engineering, and Biomedical Engineering, Yong Loo Lin School of Medicine and College of Design and Engineering, National University of Singapore, Singapore, 119074, Singapore

[3]Multi-Scale Robotics Lab (MSRL), Institute of Robotics & Intelligent Systems (IRIS), ETH Zurich, Zurich 8092, Switzerland

[4]School of Pharmacy, Shenyang Pharmaceutical University, Shenyang Liaoning, 110016, China

[5]School of Medicine, South China University of Technology, Guangzhou 510006, P.R. China.

[6]Clinical Imaging Research Centre, Centre for Translational Medicine, Yong Loo Lin School of Medicine, National University of Singapore, Singapore 117599, Singapore

[7]Nanomedicine Translational Research Program, Yong Loo Lin School of Medicine, National University of Singapore, Singapore 117597, Singapore

[8]Institute of Molecular and Cell Biology, Agency for Science, Technology, and Research (A*STAR), 61 Biopolis Drive, Proteos, Singapore, 138673, Singapore

[#]These authors contributed equally to this work.

[*]Corresponding authors: Zhonggui He, Qianqian Ni, Xiaoyuan Chen, and Jin Sun

E-mail address: hezhgui_student@aliyun.com (Z.H.); qqian.ni@nus.edu.sg (Q.N.); chen.shawn@nus.edu.sg (X.C.); and sunjin@syphu.edu.cn (J.S.).

Supplementary Figures

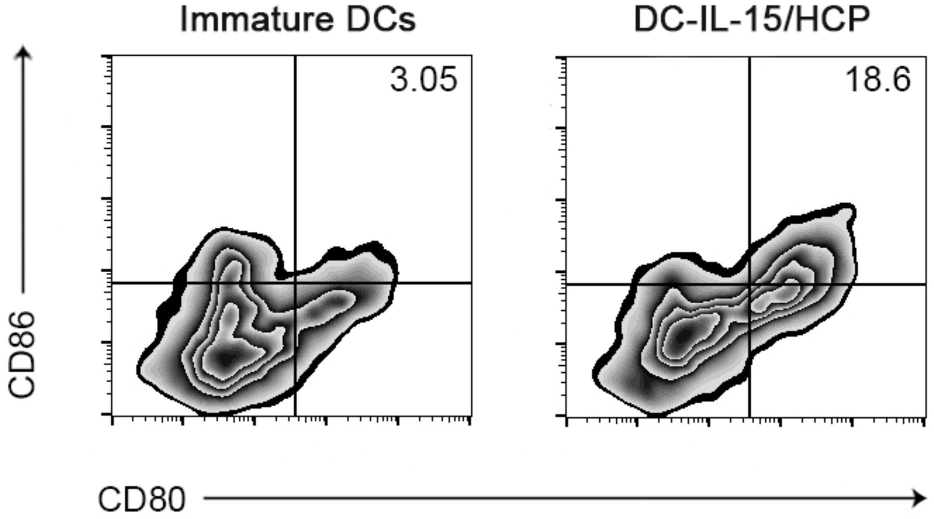

Supplementary Figure 1. Flow cytometric examination of DC-IL-15/HCP maturation. The maturation of DCs was assessed by flow cytometry gated on $CD11c^{+}CD80^{+}CD86^{+}$.

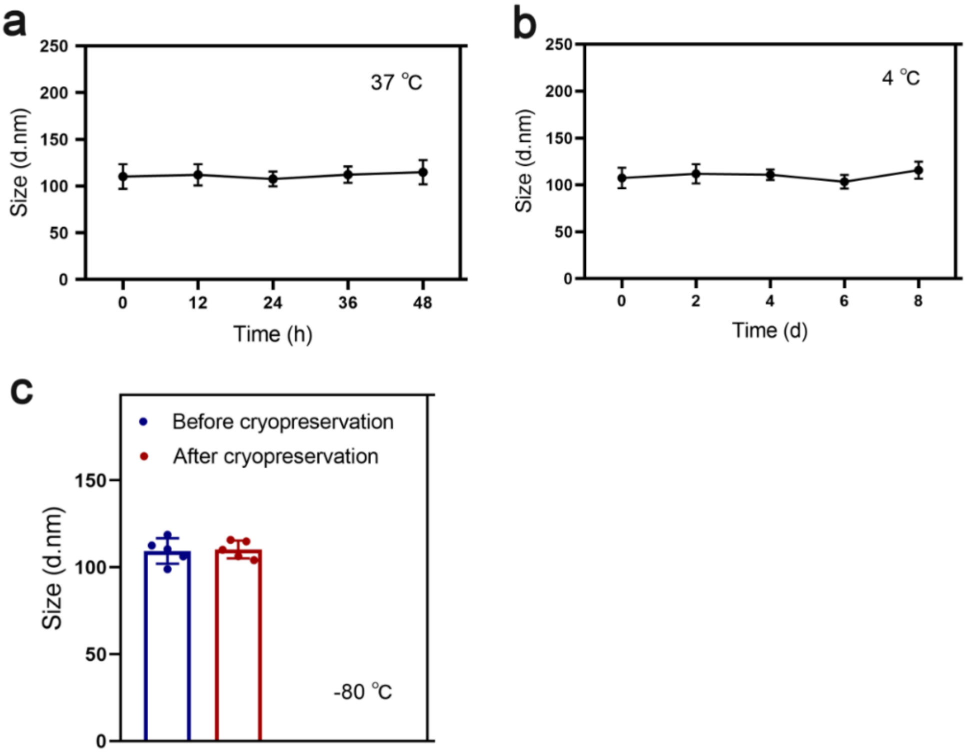

Supplementary Figure 2. **Characterization of biNV-IL-15.** biNV-IL-15 was suspended at a final concentration of 2 mg mL^{-1} in PBS. biNV-IL-15 was kept at 37 °C (**a**) or 4 °C (**b**) and particle size was measured by DLS (n = 5/group). (**c**) Diameter of biNV-IL-15 before (blue column) and after cryopreservation (red column) at -80 °C for 60 d (n = 5/group). Data represent the mean ± s.d. Source data are provided as a Source Data file.

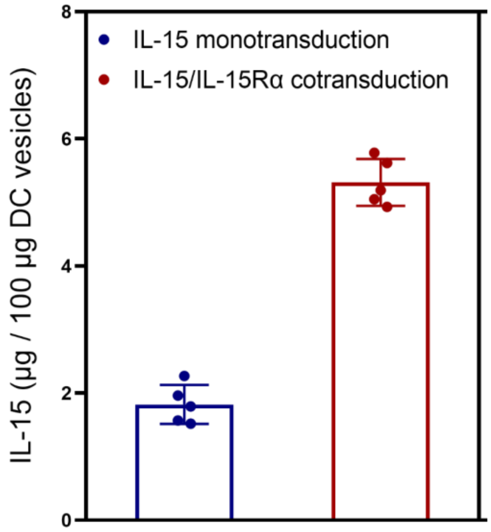

Supplementary Figure 3. The quantitative assay of IL-15 on DC vesicles. The binding efficiency could reach about 5.31 µg IL-15 per 100 µg DC vesicles (n = 5/group). Data represent the mean ± s.d. Source data are provided as a Source Data file.

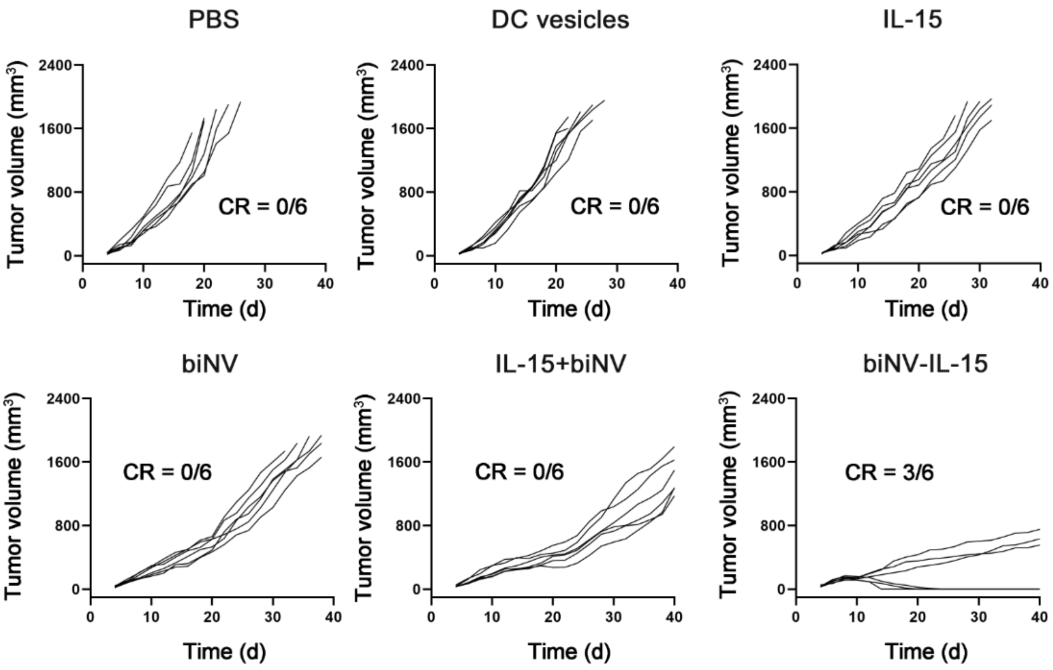

Supplementary Figure 4. Individual tumor growth curves in 4T1 tumor model receiving various treatments (n = 6), CR: complete tumor regression. Source data are provided as a Source Data file.

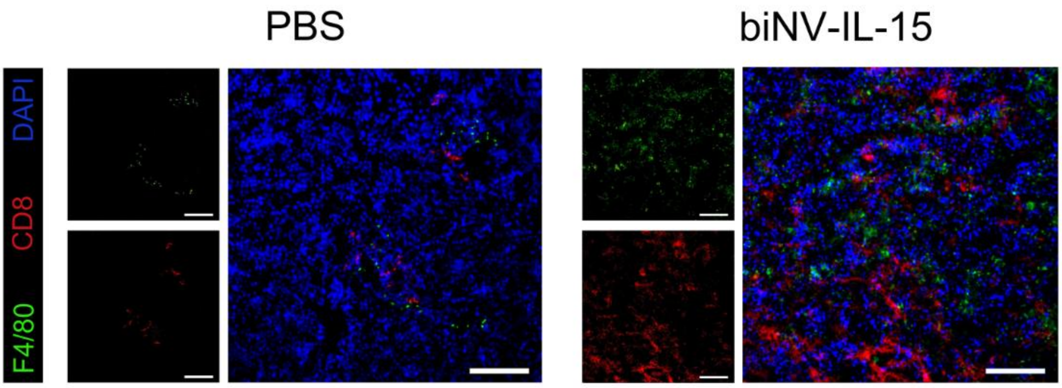

Supplementary Figure 5. Immunofluorescence images of tumors displaying F4/80^{+} macrophage and CD8^{+} T cell infiltration for PBS and biNV-IL-15 groups in 4T1 tumor model. Scale bar = 50 μm. Experiment was repeated three times independently with similar results.

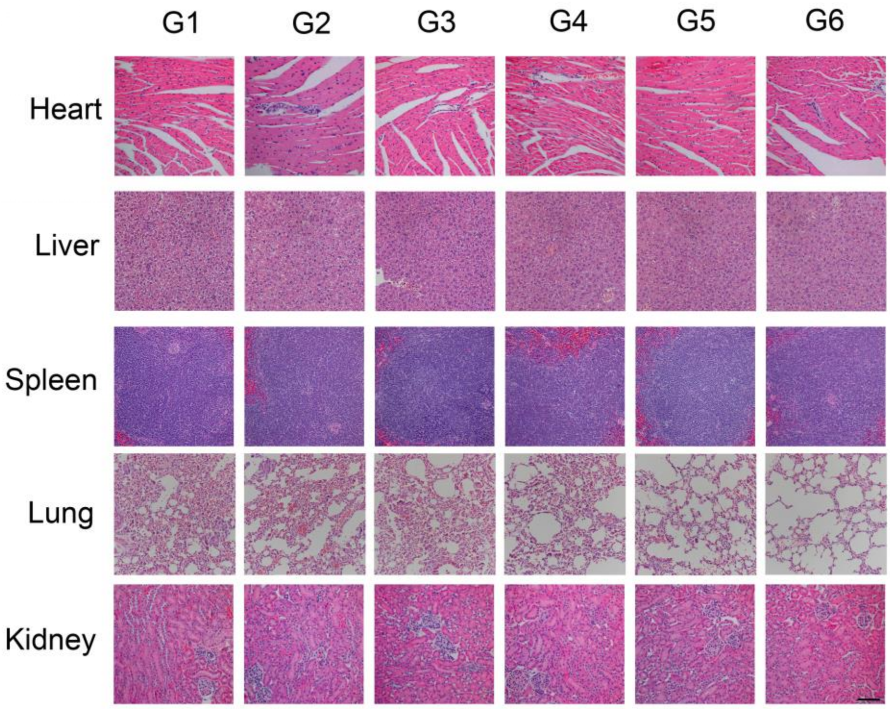

Supplementary Figure 6. H&E staining for 4T1 tumor model. G1: PBS, G2: DC vesicles, G3: IL-15, G4: biNV, G5: IL-15+biNV, G6: biNV-IL-15. H&E staining of heart, liver, spleen, lung, and kidney after various treatments. Scale bar = 100 μm. Experiment was repeated three times independently with similar results.

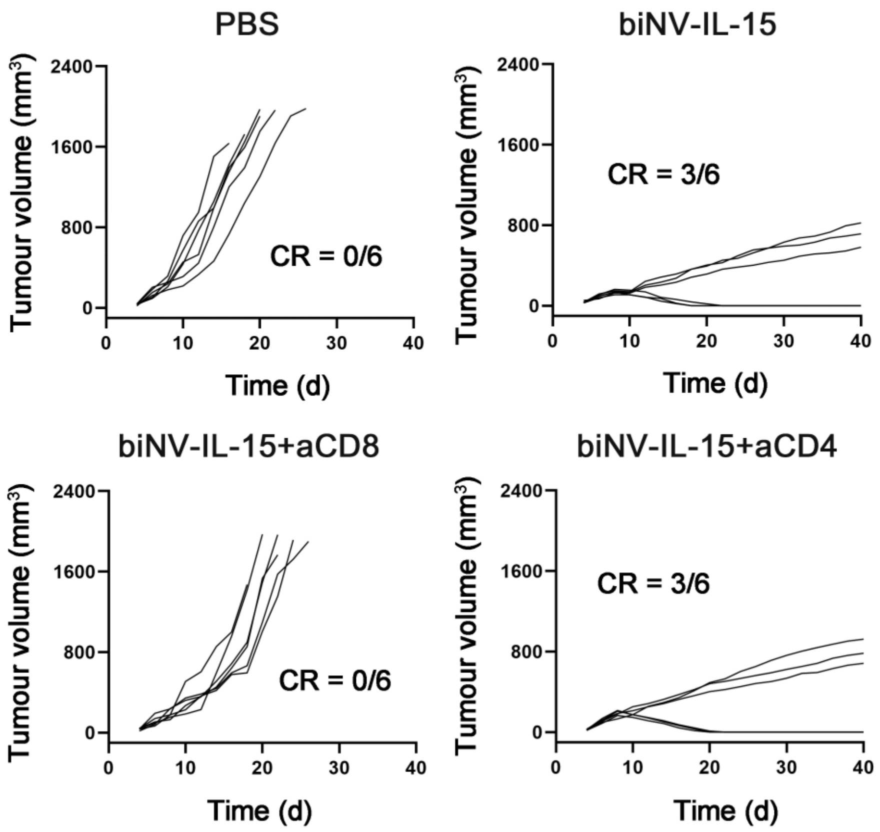

Supplementary Figure 7. Individual tumor growth curves of 4T1 tumor-bearing mice after biNV-IL-15 administration and lymphocyte depletion (n = 6), CR: complete tumor regression. Source data are provided as a Source Data file.

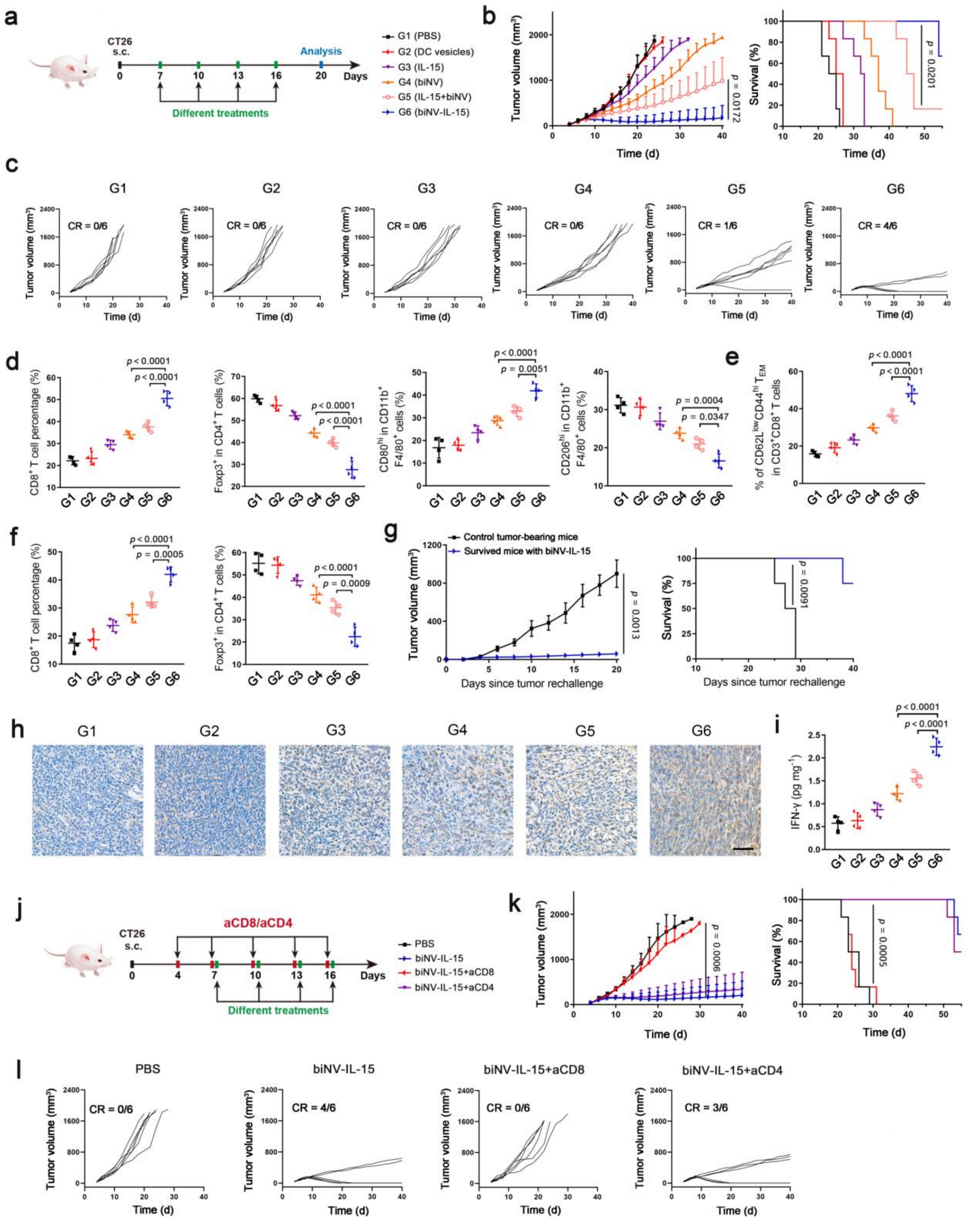

Supplementary Figure 8. **In vivo antitumor activity of biomimetic nanovaccine-mediated multivalent IL-15 self-transpresentation (MIST).** (**a**) CT26 cancer cells were subcutaneously inoculated into BALB/c mice. On days 7, 10, 13, and 16, the PBS, DC vesicles, IL-15, biNV, IL-15+biNV, and biNV-IL-15 were intravenously administrated into the mice. (**b**) Average tumor growth curve and survival curve following various treatments (n = 6/group). (**c**) Individual tumor growth curves in (**b**), CR: complete tumor regression. (**d**) Flow cytometric

quantification of tumor-infiltrating $CD8^+$ T cells, $CD4^+Foxp3^+$ Tregs, M1-type TAM ($CD80^{hi}CD11b^+F4/80^+$) and M2-type TAM ($CD206^{hi}CD11b^+F4/80^+$) following various treatments (n = 4/group). (**e**) Flow cytometric quantification of $CD3^+CD8^+CD62L^{low}CD44^{hi}$ effector memory T cells (T_{EM}) in the spleen after various treatments (n = 4/group). (**f**) Flow cytometric quantification of $CD8^+$ T cells and $CD4^+Foxp3^+$ Tregs in dLNs (n = 4/group). Survived mice in the biNV-IL-15 group on day 55 were rechallenged with CT26 cancer cells. Naive mice of the same age were implanted with cancer cells as control. (**g**) The tumor growth curve and survival curve of CT26 tumor-rechallenged mice were recorded (n = 4/group). (**h**) Immunohistochemical staining for TNF-α of tumor sections following different therapies. Scale bar = 100 μm. (**i**) The secretion of IFN-γ in the tumor was detected *via* ELISA assay (n = 4/group). (**j**) Experimental design for immune depletion in a CT26 tumor model. (**k**) Average tumor growth curve and survival curve (n = 6) of CT26 tumor-bearing mice after biNV-IL-15 administration along with lymphocyte depletion (aCD8: 100 μg, aCD4: 100 μg). (**l**) Individual tumor growth curves in (**k**) (n = 6), CR: complete tumor regression. Data represent the mean ± s.d. The *p* values of G4 to G6 in panels **d**, **e**, **f**, **i** are <0.0001, <0.0001, <0.0001, <0.0001, 0.0004, <0.0001, <0.0001, <0.0001, and <0.0001, respectively. And the *p* values of G5 to G6 in panels **d**, **e**, **f**, **i** are <0.0001, <0.0001, 0.0051, 0.0347, 0.0004, <0.0001, 0.0005, 0.0009, and <0.0001, respectively. Statistical significance was calculated through one-way ANOVA using a Tukey post-hoc test (**b**, **d-f**, **i**, **k**), log-rank (Mantel-Cox) test (**b**, **g**, **k**), or two-tailed student's t-test (**g**). Source data underlying panels **b-g**, **i**, **k**, **l** are provided as a Source Data file.

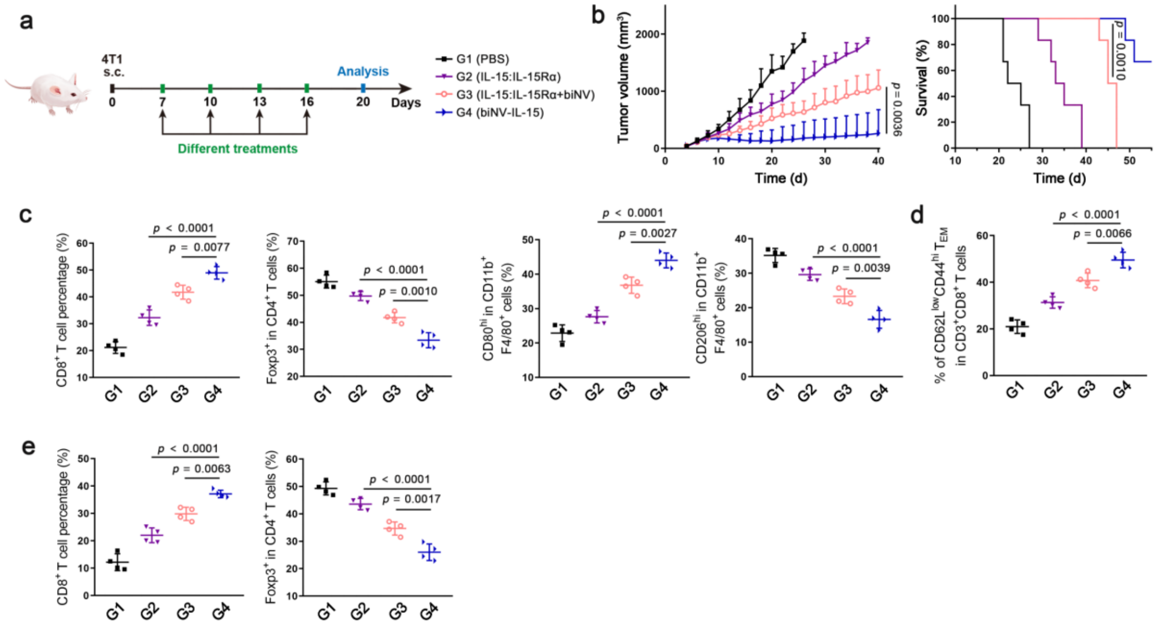

Supplementary Figure 9. In vivo antitumor activity of biomimetic nanovaccine-mediated multivalent IL-15 self-transpresentation (MIST). (**a**) 4T1 cancer cells were subcutaneously inoculated into BALB/c mice. On days 7, 10, 13, and 16, the PBS, IL-15:IL-15Rα, IL-15:IL-15Rα+biNV, and biNV-IL-15 were intravenously administered into the mice. (**b**) Average tumor growth curve and survival curve following various treatments (n = 6/group). (**c**) Flow cytometric quantification of tumor-infiltrating $CD8^+$ T cells, $CD4^+Foxp3^+$ Tregs, M1-type TAMs ($CD80^{hi}CD11b^+F4/80^+$), and M2-type TAMs ($CD206^{hi}CD11b^+F4/80^+$) in 4T1 tumor model following various treatments (n = 4/group). (**d**) Flow cytometric quantification of $CD3^+CD8^+CD62L^{low}CD44^{hi}$ effector memory T cells (T_{EM}) in the spleen after various treatments (n = 4/group). (**e**) Flow cytometric quantification of $CD8^+$ T cells and $CD4^+Foxp3^+$ Tregs in dLNs (n = 4/group). Data represent the mean ± s.d. The *p* values of G2 to G4 in panels **c**, **d**, **e** are <0.0001. And the *p* values of G3 to G4 in panels **c**, **d**, **e** are 0.0077, 0.0010, 0.0027, 0.0039, 0.0066, 0.0063, and, 0.0017, respectively. Statistical significance was calculated through two-tailed student's t-test (**b**), log-rank (Mantel-Cox) test (**b**), or one-way ANOVA using a Tukey post-hoc test (**c-e**). Source data underlying panels **b-e** are provided as a Source Data file.

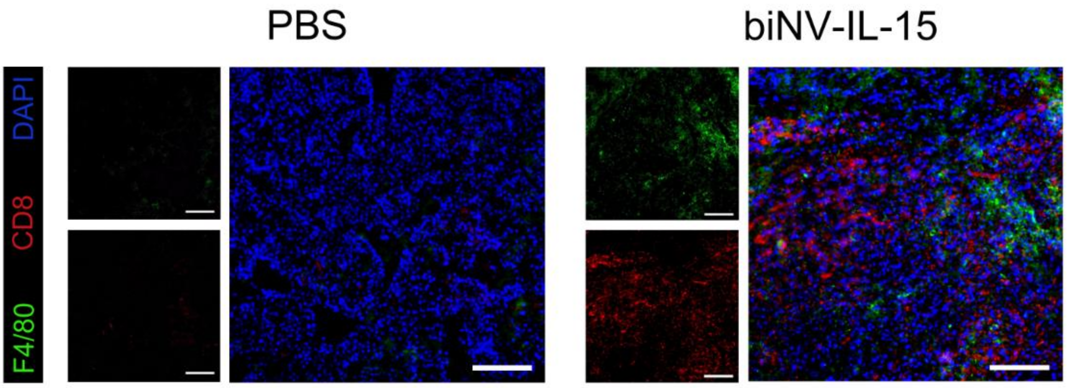

Supplementary Figure 10. Immunofluorescence images of tumors displaying F4/80$^+$ macrophage and CD8$^+$ T cell infiltration for PBS and biNV-IL-15 groups in the CT26 tumor model. Scale bar = 50 μm. Experiment was repeated three times independently with similar results.

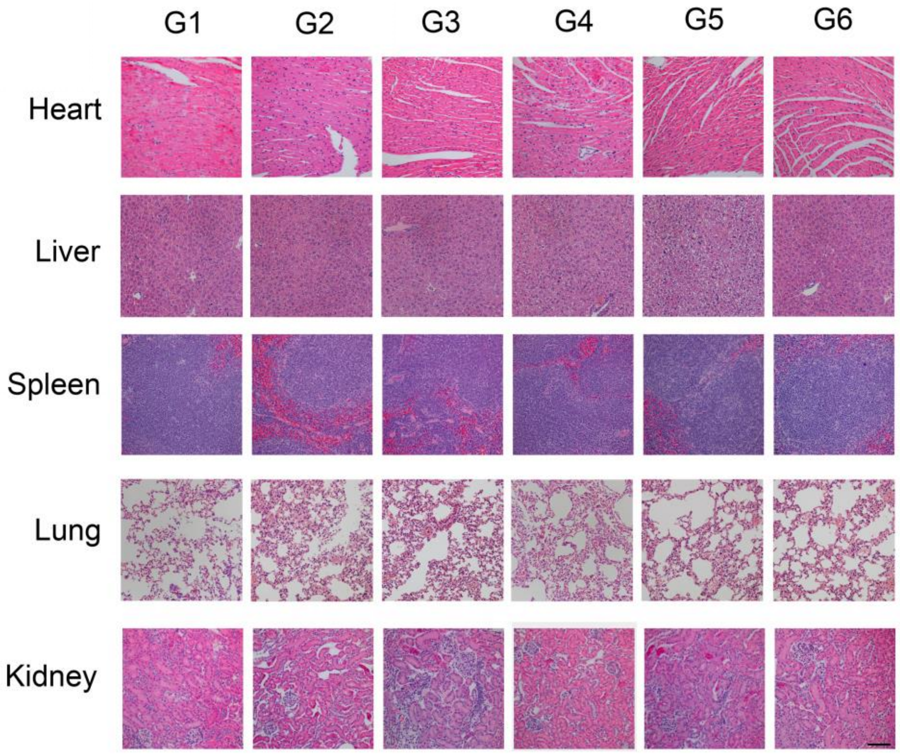

Supplementary Figure 11. H&E staining for CT26 tumor model. G1: PBS, G2: DC vesicles, G3: IL-15, G4: biNV, G5: IL-15+biNV, G6: biNV-IL-15. H&E staining of heart, liver, spleen, lung, and kidney after various treatments. Scale bar = 100 μm. Experiment was repeated three times independently with similar results.

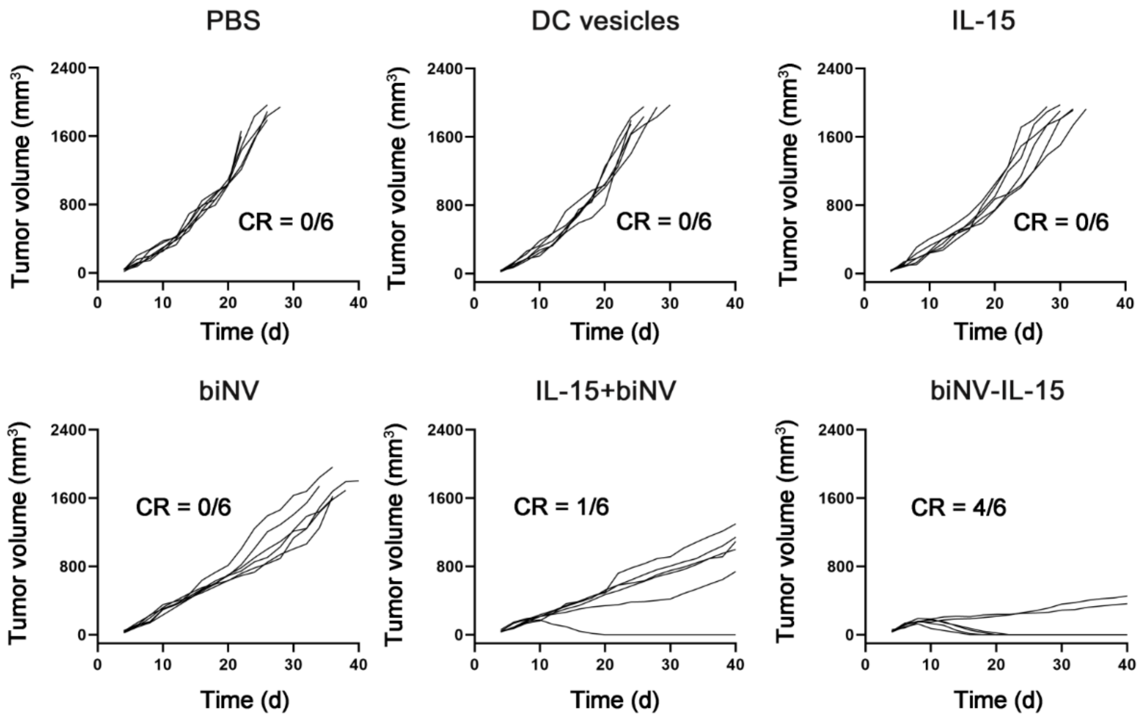

Supplementary Figure 12. Individual tumor growth curves in B16F10-OVA tumor model receiving various treatments (n = 6), CR: complete tumor regression. Source data are provided as a Source Data file.

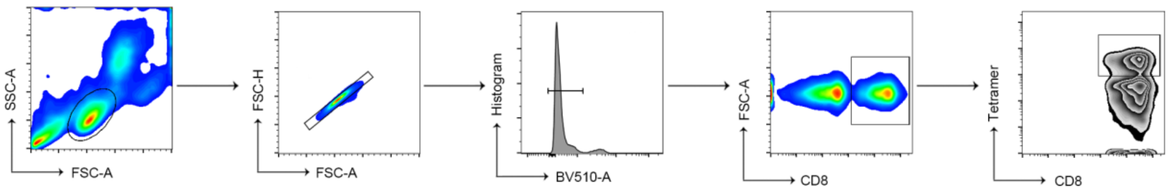

Supplementary Figure 13. Gating strategy for the flow cytometry analysis of SIINFEKL-specific $CD8^+$ T cells in peripheral blood of B16F10-OVA tumor-bearing mice after various treatments, which was monitored through flow cytometry analysis of $tetramer^+CD8^+$ T cells (Figure 6c).

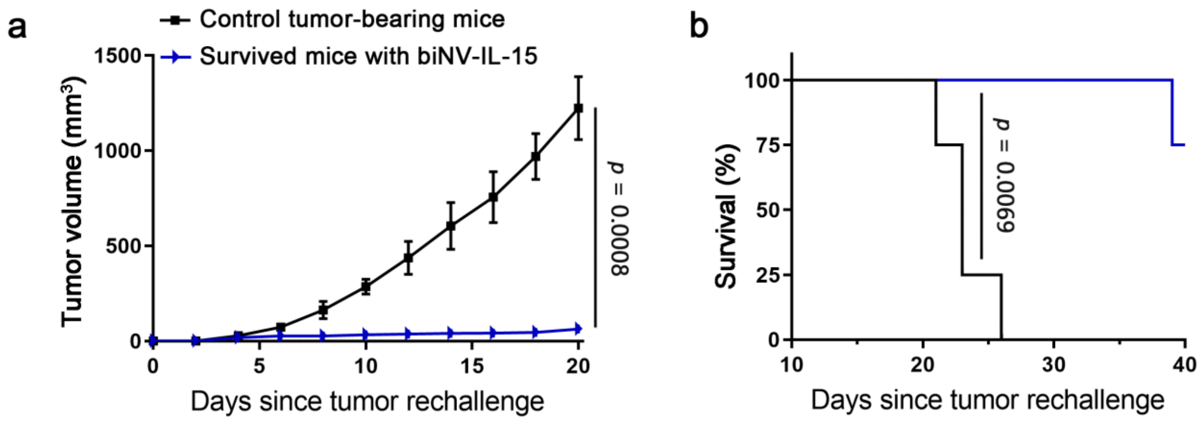

Supplementary Figure 14. Survived mice in the biNV-IL-15 group of the B16F10-OVA tumor model on day 55 were rechallenged with B16F10-OVA cancer cells. Naive mice of the same age were implanted with cancer cells as control. (**a**) Tumor growth curve and (**b**) survival curve of rechallenged mice were recorded (n = 4/group). Data represent the mean ± s.d. Statistical significance was calculated through two-tailed student's t-test (**a**) or log-rank (Mantel-Cox) test (**b**). Source data underlying panels **a**, **b** are provided as a Source Data file.

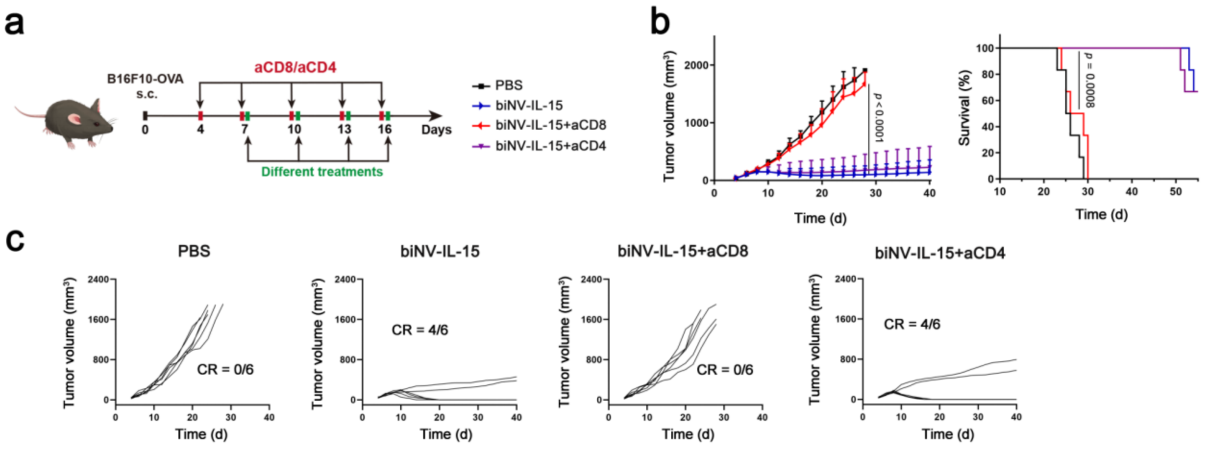

Supplementary Figure 15. (**a**) Experimental design for immune depletion in B16F10-OVA tumor model. (**b**) Average tumor growth curve and survival curve (n = 6) of B16F10-OVA tumor-bearing mice after biNV-IL-15 administration along with lymphocyte depletion (aCD8: 100 μg, aCD4: 100 μg). (**c**) Individual tumor growth curves in (**b**), CR: complete tumor regression. Data represent the mean ± s.d. The *p* values of biNV-IL-15 to biNV-IL-15+aCD8 in panel **b** are <0.0001 and 0.0008, respectively. Statistical significance was calculated through one-way ANOVA using a Tukey post-hoc test (**b**) or log-rank (Mantel-Cox) test (**b**). Source data underlying panels **b**, **c** are provided as a Source Data file.

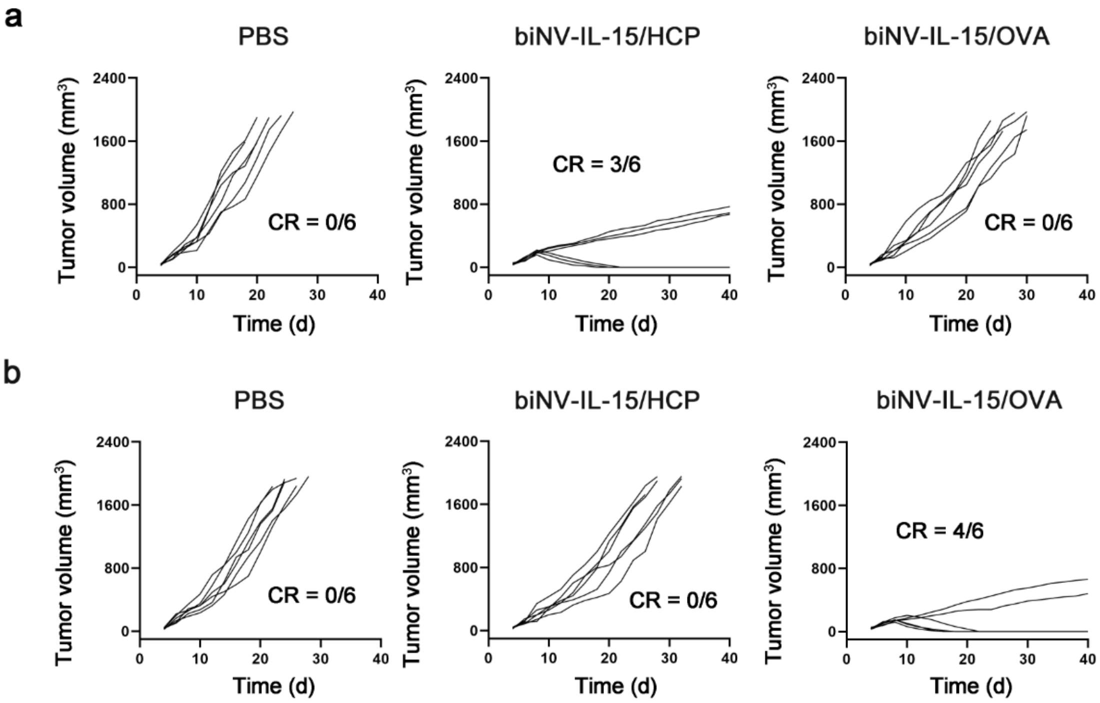

Supplementary Figure 16. Individual tumor growth curve of 4T1 (**a**) or B16F10-OVA (**b**) tumor-bearing mice suggested the in vivo antigen-specific cancer inhibition of biNV-IL-15 (n = 6), CR: complete tumor regression. Source data are provided as a Source Data file.

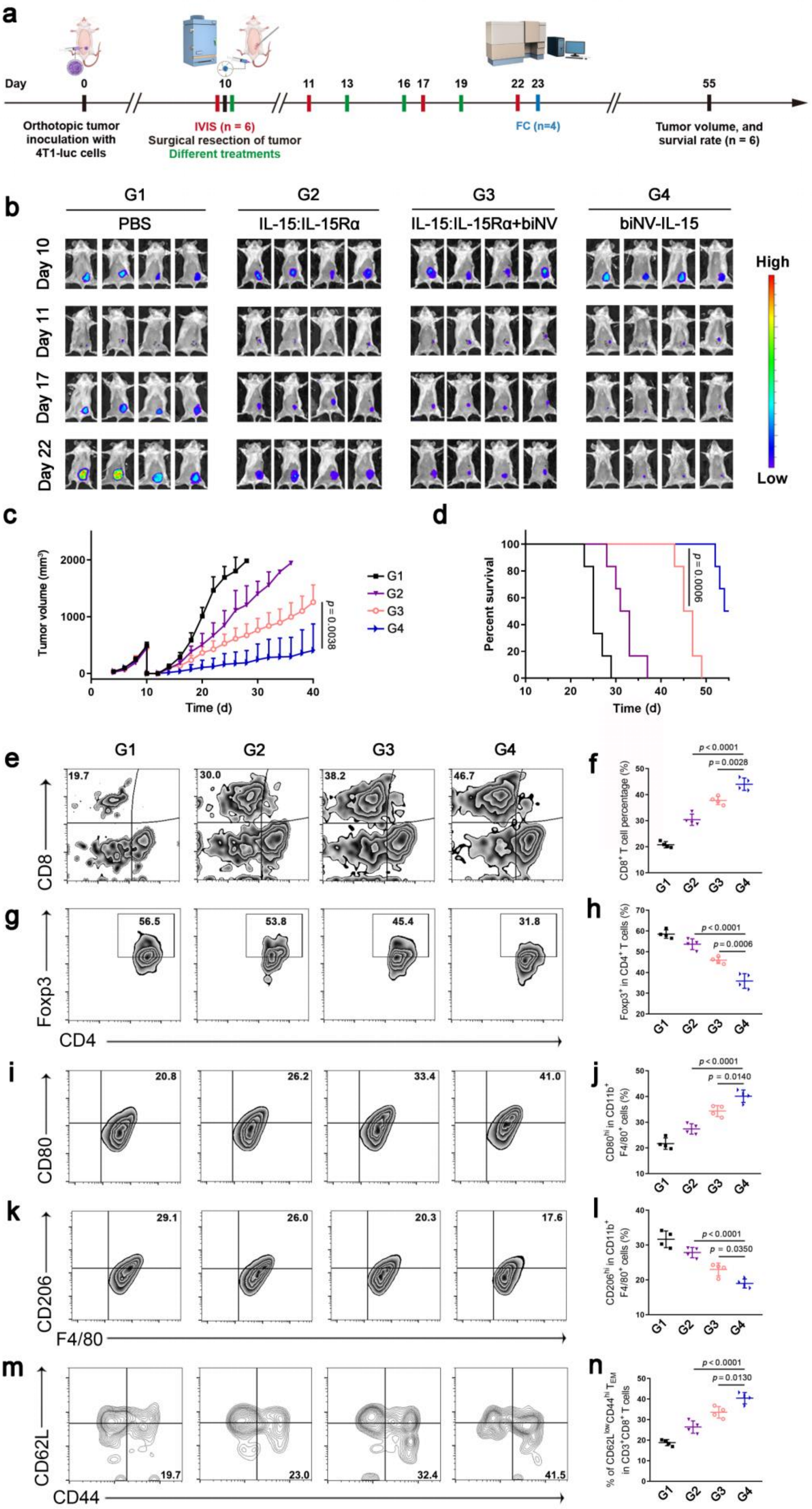
a
Day
0
10
11
13
16
17
19
22
23
55
Orthotopic tumor inoculation with 4T1-luc cells
IVIS (n = 6)
Surgical resection of tumor
Different treatments
FC (n=4)
Tumor volume, and survial rate (n = 6)
b
G1
PBS
G2
IL-15:IL-15Rα
G3
IL-15:IL-15Rα+biNV
G4
biNV-IL-15
Day 10
Day 11
Day 17
Day 22
High
Low
c
Tumor volume (mm³)
Time (d)
p = 0.0038
d
Percent survival
Time (d)
p = 0.0006
e
CD8
f
CD8⁺ T cell percentage (%)
p < 0.0001
p = 0.0028
g
Foxp3
CD4
h
Foxp3⁺ in CD4⁺ T cells (%)
p < 0.0001
p = 0.0006
i
CD80
j
CD80ʰⁱ in CD11b⁺ F4/80⁺ cells (%)
p < 0.0001
p = 0.0140
k
CD206
F4/80
l
CD206ʰⁱ in CD11b⁺ F4/80⁺ cells (%)
p < 0.0001
p = 0.0350
m
CD62L
CD44
n
% of CD62Lˡᵒʷ CD44ʰⁱ T_EM in CD3⁺CD8⁺ T cells
p < 0.0001
p = 0.0130

Supplementary Figure 17. The suppression of tumor recurrence. (**a**) Schematic depicting biNV-IL-15 treatment in 4T1-luc orthotopic cancer model with incomplete cancer resection (IVIS: in vivo imaging system; FC: flow cytometry analysis). (**b**) In vivo bioluminescence imaging for 4T1-luc tumor following the removal of the primary tumor. Every group showed four representative mice. Images on day 10 were shown before surgery. (**c**) Average tumor growth curve and (**d**) survival curve (n = 6/group) in tumor resection model receiving various treatments. (**e**) Flow cytometric examination images and (**f**) relative quantification of $CD8^+$ T cell in tumor (n = 4/group). (**g**) Flow cytometric measurement images and (**h**) relative quantification of $CD4^+Foxp3^+$ Tregs in tumor (n = 4/group). (**i**) Flow cytometric assessment images and (**j**) relative quantification of M1-type TAMs ($CD80^{hi}CD11b^+F4/80^+$) in tumor (n = 4/group). (**k**) Flow cytometric analysis images and (**l**) relative quantification of M2-type TAMs ($CD206^{hi}CD11b^+F4/80^+$) in tumor (n = 4/group). (**m**) Flow cytometric evaluation images and (**n**) relative quantification of $CD3^+CD8^+CD62L^{low}CD44^{hi}$ T_{EM} in the spleen (n = 4/group). Data represent the mean ± s.d. The *p* values of G2 to G4 in panels **f**, **h**, **j**, **l**, **n** are <0.0001. And the *p* values of G3 to G4 in panels **f**, **h**, **j**, **l**, **n** are 0.0028, 0.0006, 0.0140, 0.0350, and 0.0130, respectively. Statistical significance was calculated through two-tailed student's t-test (**c**), log-rank (Mantel-Cox) test (**d**), or one-way ANOVA using a Tukey post-hoc test (**f**, **h**, **j**, **l**, **n**). Source data underlying panels **c**, **d**, **f**, **h**, **j**, **l**, **n** are provided as a Source Data file.

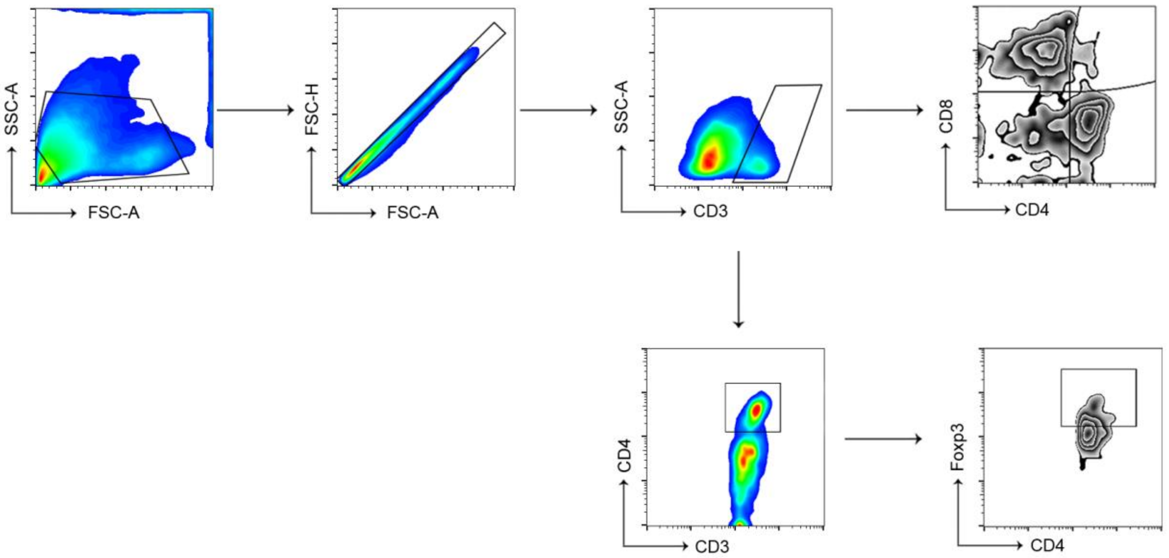

Supplementary Figure 18. Gating strategy for the flow cytometry analysis of tumor-infiltrating $CD8^+$ T cells and $CD4^+Foxp3^+$ Tregs (Figure 7g, i).

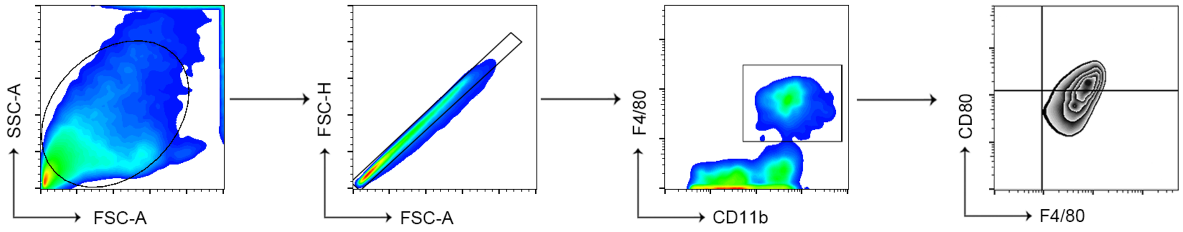

Supplementary Figure 19. Gating strategy for the flow cytometry analysis of M1-like macrophages ($CD80^{hi}CD11b^{+}F4/80^{+}$) in tumor (Figure 7k).

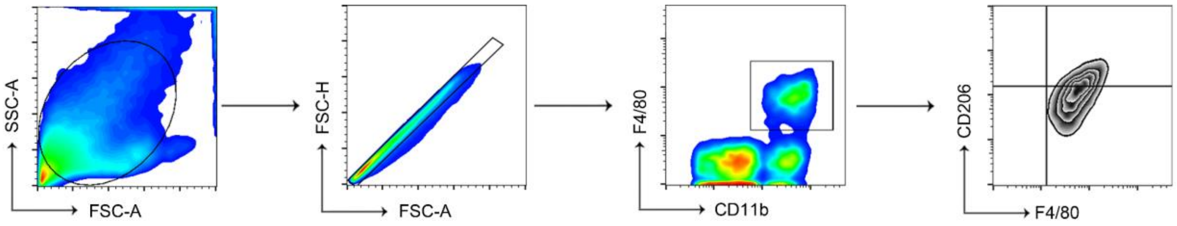

Supplementary Figure 20. Gating strategy for the flow cytometry analysis of M2-like macrophages (CD206^{hi}CD11b^{+}F4/80^{+}) in tumor (Figure 7m).

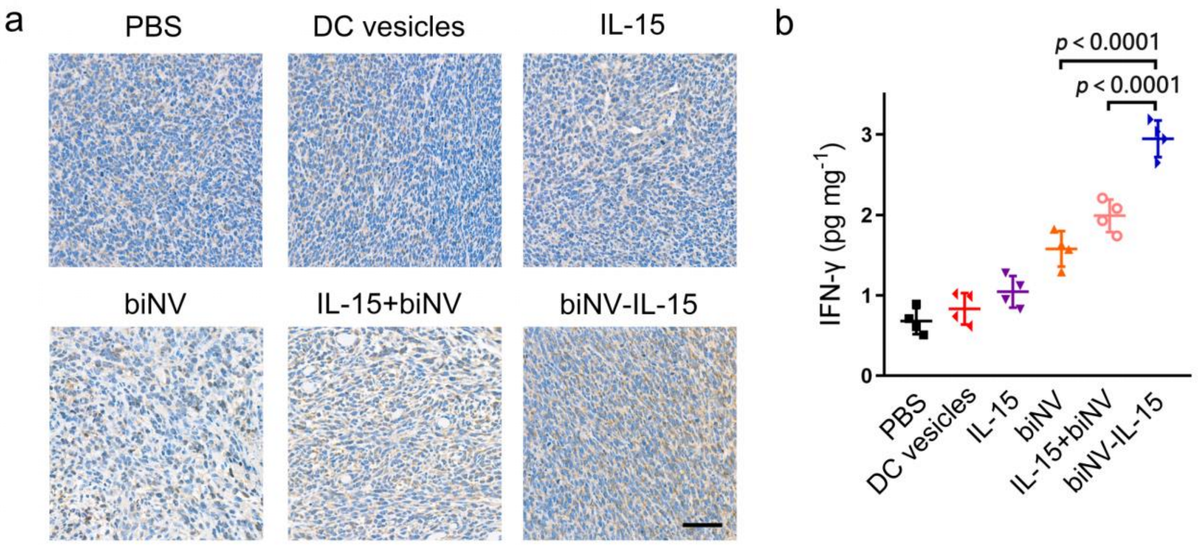

Supplementary Figure 21. (**a**) Immunohistochemical staining for TNF-α of tumor sections in tumor resection model following different therapies. Scale bar = 100 μm. (**b**) The secretion of IFN-γ in the tumor was detected *via* ELISA assay (n = 4/group). Data represent the mean ± s.d. The *p* values in panel **b** are <0.0001. Statistical significance was calculated through one-way ANOVA using a Tukey post-hoc test. Source data underlying panel **b** are provided as a Source Data file.

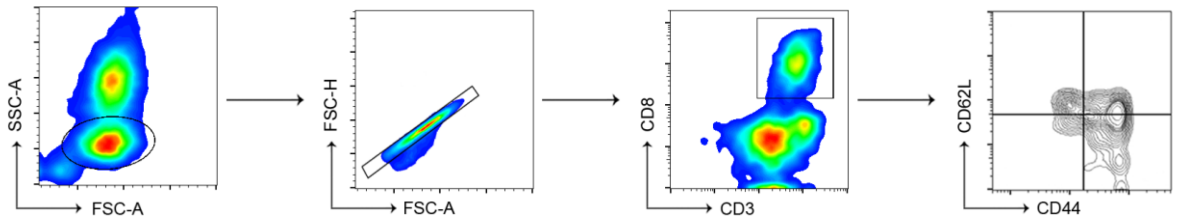

Supplementary Figure 22. Gating strategy for the flow cytometry analysis of $CD3^{+}CD8^{+}CD62L^{low}CD44^{hi}$ T_{EM} in spleen (Figure 7o).

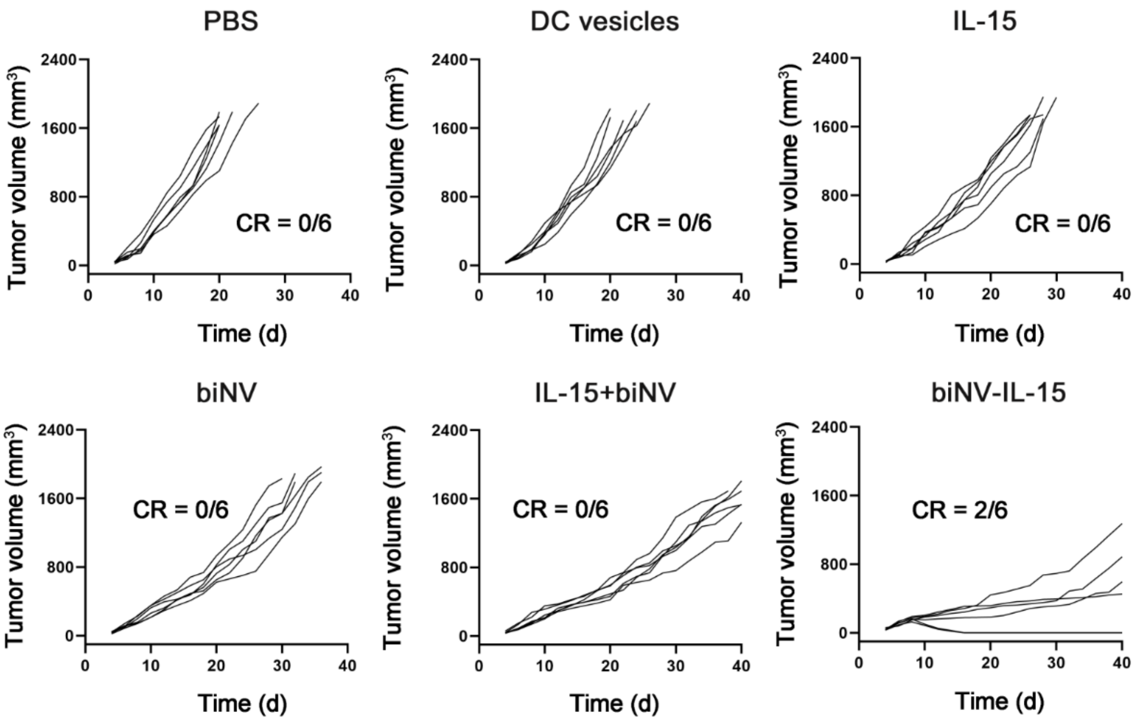

Supplementary Figure 23. Individual tumor growth curves in hematogenous metastasis model receiving various treatments (n = 6), CR: complete tumor regression. Source data are provided as a Source Data file.

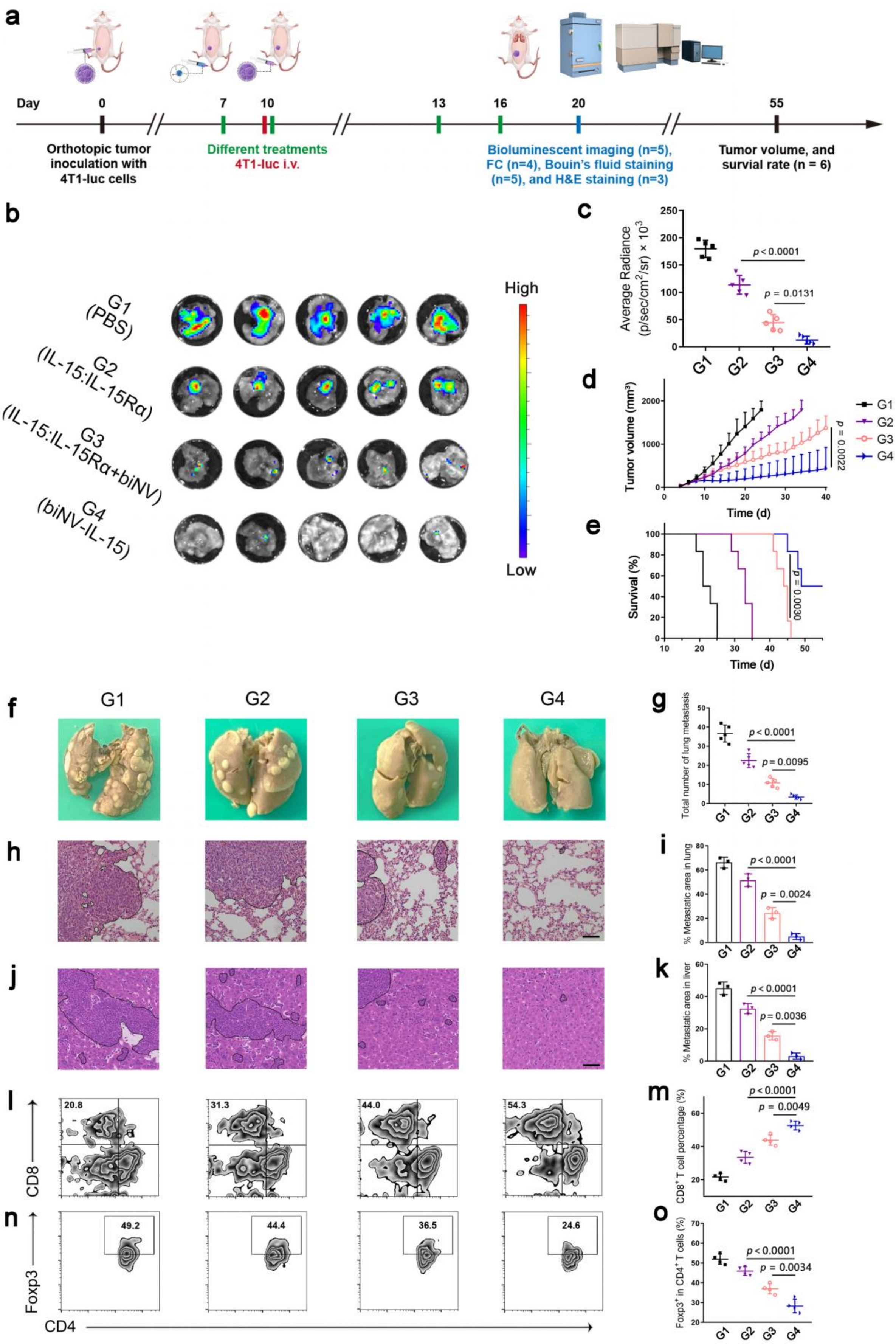

Supplementary Figure 24. In vivo anti-metastasis performance. (**a**) 4T1-luc cancer cells

were intravenously injected into the tumor-bearing mice on day 10 to simulate the hematogenous metastasis model (FC: flow cytometry analysis; H&E staining: hematoxylin and eosin staining). PBS, IL-15:IL-15Rα, IL-15:IL-15Rα+biNV, and biNV-IL-15 were individually administrated on days 7, 10, 13, and 16. (**b**) Ex vivo bioluminescent imaging and (**c**) average bioluminescent radiance for isolated lungs were studied after various treatments on day 20 (n = 5/group). (**d**) Average tumor growth curve and (**e**) survival curve (n = 6/group) in hematogenous metastasis model receiving various treatments. (**f**) Photographs and (**g**) quantification of lung metastases (n = 5/group) for Bouin's fluid staining of lungs. H&E staining of the lung (**h**) and liver (**j**) slices after different treatments. Scale bar = 100 μm. Quantification for metastasis area percentages of the lung (**i**) and liver (**k**) slices (n = 3/group). (**l**) Flow cytometric analysis images and (**m**) relative quantification of $CD8^+$ T cells in the blood (n = 4/group). (**n**) Flow cytometric examination images and (**o**) relative quantification of $CD4^+Foxp3^+$ Tregs in the blood (n = 4/group). Data represent the mean ± s.d. The *p* values of G2 to G4 in panels **c**, **g**, **i**, **k**, **m**, **o** are <0.0001. And the *p* values of G3 to G4 in panels **c**, **g**, **i**, **k**, **m**, **o** are 0.0131, 0.0095, 0.0024, 0.0036, 0.0049, and 0.0034, respectively. Statistical significance was calculated through one-way ANOVA using a Tukey post-hoc test (**c**, **g**, **i**, **k**, **m**, **o**), two-tailed student's t-test (**d**) or log-rank (Mantel-Cox) test (**e**). Source data underlying panels **c-e**, **g**, **i**, **k**, **m**, **o** are provided as a Source Data file.
