# Supplementary material for: Biomimetic nanovaccine-mediated multivalent IL-15 self-transpresentation (MIST) for potent and safe cancer immunotherapy
Source: Nat Commun. 2023 Oct 24;14:6748. doi: 10.1038/s41467-023-42155-z (PMC10598200; doi:10.1038/s41467-023-42155-z)
Supplement: Supplementary file 3 — Reporting Summary [file 41467_2023_42155_MOESM3_ESM.pdf]

nature portfolio

Corresponding author(s): Xiaoyuan Chen

Last updated by author(s): Sep 1, 2023

Reporting Summary

Nature Portfolio wishes to improve the reproducibility of the work that we publish. This form provides structure for consistency and transparency in reporting. For further information on Nature Portfolio policies, see our Editorial Policies and the Editorial Policy Checklist.

Statistics

For all statistical analyses, confirm that the following items are present in the figure legend, table legend, main text, or Methods section.

n/a	Confirmed	
☐	☒	The exact sample size (n) for each experimental group/condition, given as a discrete number and unit of measurement
☐	☒	A statement on whether measurements were taken from distinct samples or whether the same sample was measured repeatedly
☐	☒	The statistical test(s) used AND whether they are one- or two-sided<br>*Only common tests should be described solely by name; describe more complex techniques in the Methods section.*
☒	☐	A description of all covariates tested
☐	☒	A description of any assumptions or corrections, such as tests of normality and adjustment for multiple comparisons
☐	☒	A full description of the statistical parameters including central tendency (e.g. means) or other basic estimates (e.g. regression coefficient) AND variation (e.g. standard deviation) or associated estimates of uncertainty (e.g. confidence intervals)
☐	☒	For null hypothesis testing, the test statistic (e.g. F, t, r) with confidence intervals, effect sizes, degrees of freedom and P value noted<br>*Give P values as exact values whenever suitable.*
☒	☐	For Bayesian analysis, information on the choice of priors and Markov chain Monte Carlo settings
☒	☐	For hierarchical and complex designs, identification of the appropriate level for tests and full reporting of outcomes
☒	☐	Estimates of effect sizes (e.g. Cohen's d, Pearson's r), indicating how they were calculated

Our web collection on statistics for biologists contains articles on many of the points above.

Software and code

Policy information about availability of computer code

Data collection	Malvern: Zetasizer Software 7.01; Microplate reader: SkanIt 2.4.3.37; Confocal laser scanning microscopy: NIS 4.13; Flow cytometer: BD CellQuest Pro v5.2
Data analysis	Gene Mapper ID 3.2 software; GraphPad prism 8.0; Flowjo_V10; DAS 2.1.1; Living image software (Perkin Elmer); Image J (v1.8.0); AID ELISPOT software (version 5.0)

For manuscripts utilizing custom algorithms or software that are central to the research but not yet described in published literature, software must be made available to editors and reviewers. We strongly encourage code deposition in a community repository (e.g. GitHub). See the Nature Portfolio guidelines for submitting code & software for further information.

Data

Policy information about availability of data

All manuscripts must include a data availability statement. This statement should provide the following information, where applicable:

- Accession codes, unique identifiers, or web links for publicly available datasets
- A description of any restrictions on data availability
- For clinical datasets or third party data, please ensure that the statement adheres to our policy

All data supporting the findings of this study are available within the Article, Supplementary Information or Source Data file. The source data underlying Figure 2g, i, Figure 3c-i, k, Figure 4b-h, Figure 5b-f, h, j, Figure 6b, d-f, Figure 7c-e, h, j, l, n, p, Figure 8c-e, g, i, k, m, o, supplementary Figures 2, 3, 4, 7, 8b-g, i, k, l, 9b-e, 12, 16a, b, 14a, b, 15b, c, 17c, d, f, h , j, l, n, 21b, 23, 24c-e, g, i, k, m, o have been deposited in the Figshare database (https://doi.org/10.6084/m9.figshare.24079164).

Human research participants

Policy information about studies involving human research participants and Sex and Gender in Research.

Reporting on sex and gender	not applicable
Population characteristics	not applicable
Recruitment	not applicable
Ethics oversight	not applicable

Note that full information on the approval of the study protocol must also be provided in the manuscript.

Field-specific reporting

Please select the one below that is the best fit for your research. If you are not sure, read the appropriate sections before making your selection.

[x] Life sciences [ ] Behavioural & social sciences [ ] Ecological, evolutionary & environmental sciences

For a reference copy of the document with all sections, see nature.com/documents/nr-reporting-summary-flat.pdf

Life sciences study design

All studies must disclose on these points even when the disclosure is negative.

Sample size	Sample size was selected from the general sample size in the reference. Increasing the number of parallelisms could reduce accidental error and improve the precision of the experiment. According to the statistical principle, when 'n' is increased, the corresponding precision will also be improved. For property measurement experiments, samples were prepared and tested at least twice. For in vivo studies, each group contains at least 3 (n ≥3) for evaluating the statistical significance. A precise value of 'n' was provided in the legends of figures. Sample size was chosen in consideration of animal individual differences and the 4R principle for the credibility of the results.
Data exclusions	No data was excluded from studies.
Replication	We confirmed that all repeated attempts were successful. Experiment repeat numbers are reported in Figure Legends.
Randomization	Samples were randomly allocated into experimental groups.
Blinding	The investigators were blinded to group allocation during data collection and analysis.

Reporting for specific materials, systems and methods

We require information from authors about some types of materials, experimental systems and methods used in many studies. Here, indicate whether each material, system or method listed is relevant to your study. If you are not sure if a list item applies to your research, read the appropriate section before selecting a response.

Materials & experimental systems

n/a	Involved in the study
☐	☒ Antibodies
☐	☒ Eukaryotic cell lines
☒	☐ Palaeontology and archaeology
☐	☒ Animals and other organisms
☒	☐ Clinical data
☒	☐ Dual use research of concern

Methods

n/a	Involved in the study
☒	☐ ChIP-seq
☐	☒ Flow cytometry
☒	☐ MRI-based neuroimaging

Antibodies

Antibodies used

The following antibodies were used for immunoprecipitation, western blot, and immunolabeling. They are listed as antigen first, followed by supplier, catalog number and clone/ lot number as applicable.
1) Anti-mouse HSP-70, Santa Cruz, cat. no. sc-24;
2) Anti-mouse Na/K ATPase, Abcam, cat. no. ab254025, dilution: 1:1000;
3) Anti-mouse B7-1, Bioss, cat. no. bs-1479R, dilution: 1:1000;
4) Anti-mouse B7-2, ABclonal, cat. no. A19026, dilution: 1:1000;
5) Anti-mouse ICAM-1, Invitrogen, cat. no. MA5407, dilution: 1:250;
6) Anti-mouse CCR7, Invitrogen, cat. no. MA1-163, dilution: 1:500;
7) Anti-mouse IL-15, Abcam, cat. no. ab273625, dilution: 1:1000;
8) Anti-mouse IL-15Rα, Invitrogen, cat. no. PA5-114215, dilution: 1:500;
9) Anti-mouse MHC-I, Abcam, cat. no. ab281901, dilution: 1:100;
10) Goat Anti-Rabbit IgG H&L / Gold antibody, Bioss, cat. no. bs-0295G-Gold, dilution: 1:100;

The following antibodies were used for in vivo lymphocyte depletion. They are listed as antigen first, followed by supplier, catalog number and clone/lot number as applicable.
1) InVivoPlus anti-mouse CD4, BioXCell, cat. no. BP0003-1, clone: GK1.5;
2) InVivoPlus anti-mouse CD8α, BioXCell, cat. no. BP0061, clone: 2.43;

The following antibodies were used for flow cytometry. They are listed as antigen first, followed by supplier, catalog number and clone/lot number as applicable.
1) Anti-mouse CD11c, Biolegend, cat. no. 117306, Clone: N418, 1: 200 dilution;
2) Anti-mouse CD80, Biolegend, cat. no. 104707, Clone: 16-10A1, 1: 200 dilution;
3) Anti-mouse CD86, Biolegend, cat. no. 105011, Clone: GL-1, 1: 200 dilution;
4) Anti-mouse CD11b, Biolegend, cat. no. 101208, Clone: M1/70, 1: 200 dilution;
5) Anti-mouse F4/80, Biolegend, cat. no. 123116, Clone: BM8, 1: 200 dilution;
6) Anti-mouse CD80, Biolegend, cat. no. 104722, Clone: 16-10A1, 1: 200 dilution;
7) Anti-mouse CD206, Biolegend, cat. no. 141716, Clone: C068C2, 1: 200 dilution;
8) Anti-mouse CD3, Biolegend, cat. no. 100204, Clone: 17A2, 1: 200 dilution;
9) Anti-mouse CD4, Biolegend, cat. no. 100432, Clone: GK1.5, 1: 200 dilution;
10) Anti-mouse CD8a, Biolegend, cat. no. 100712, Clone: 53-6.7, 1: 200 dilution;
11) Anti-mouse Foxp3, Biolegend, cat. no. 126404, Clone: MF-14, 1: 200 dilution;
12) Anti-mouse CD3, Biolegend, cat. no. 100218, Clone: 17A2, 1: 200 dilution;
13) Anti-mouse CD62L, Biolegend, cat. no. 104428, Clone: MEL-14, 1: 200 dilution;
14) Anti-mouse CD8a, Biolegend, cat. no. 100706, Clone: 53-6.7, 1: 200 dilution;
15) Anti-mouse CD44, Biolegend, cat. no. 103008, Clone: IM7, 1: 200 dilution;
16) Anti-mouse CD16/32, Biolegend, cat. no. 101302, Clone: 93, 1: 200 dilution;
17) Anti-mouse SIINFEKL/H-2Kb, eBioscience, cat. no. 12-5743-81, Clone: 25-D1.16, 1: 400 dilution;

The following antibodies were used for immunohistochemistry assay. They are listed as antigen first, followed by supplier, catalog number and clone/lot number as applicable.
1) Anti-mouse HSP70, Santa Cruz, cat. no. sc-24, Clone: W27, 1: 200 dilution;
2) Anti-mouse TNF-α, Abcam, cat. no. ab1793, Clone: 52B83, 1: 40 dilution;

The following antibodies were used for immunofluorescence staining. They are listed as antigen first, followed by supplier, catalog number and clone/lot number as applicable.
1) Anti-mouse F4/80, Abcam, cat. no. ab100790, 1: 200 dilution;
2) Anti-mouse CD8 alpha, Abcam, cat. no. ab22378, 1: 200 dilution;
3) Goat anti-rabbit IgG, H+L, Thermo Fisher Scientific, cat. no. A32733, 1: 1000 dilution;
4) Goat anti-rat IgG, H+L, Thermo Fisher Scientific, cat. no. A18866, 1: 600 dilution;

Validation

The species and application of the following antibodies used for immunoprecipitation, western blot, and immunolabeling were validated by the manufacturer.
1) Anti-mouse HSP-70, Santa Cruz, https://www.scbt.com/p/hsp-70-hsc-70-antibody-w27?requestFrom=search;
2) Anti-mouse Na/K ATPase, Abcam, https://www.abcam.cn/products/panels/plasma-membrane-marker-nak-atpase-pan-cadherin-pmca1-antibody-sampler-panel-human-mouse-ab254025.html;

3) Anti-mouse B7-1, Bioss, http://www.bioss.com.cn/prolook_03.asp?id=AF08169606001258&pro37=1;
4) Anti-mouse B7-2, ABclonal, https://abclonal.com.cn/catalog/A19026;
5) Anti-mouse ICAM-1, Invitrogen, https://www.thermofisher.cn/cn/zh/antibody/product/ICAM-1-Antibody-clone-1A29-Monoclonal/MA5407;
6) Anti-mouse CCR7, Invitrogen, https://www.thermofisher.cn/cn/zh/antibody/product/CCR7-Antibody-clone-4B12-Monoclonal/MA1-163;
7) Anti-mouse IL-15, Abcam, https://www.abcam.cn/products/primary-antibodies/il-15-antibody-epr22635-214-ab273625.html;
8) Anti-mouse IL-15Rα, Invitrogen, https://www.thermofisher.cn/cn/zh/antibody/product/CD215-IL-15Ra-Antibody-Polyclonal/PA5-114215;
9) Anti-mouse MHC-I, Abcam, https://www.abcam.cn/products/primary-antibodies/mhc-class-i-antibody-r1-96-rabbit-igg-chimeric-ab281901.html;
10) Goat Anti-Rabbit IgG H&L / Gold antibody, Bioss, http://www.bioss.com.cn/prolook_03.asp?id=AF08169606011041&pro37=4;

The species and application of the following antibodies used for in vivo lymphocyte depletion were validated by the manufacturer.
1) InVivoPlus anti-mouse CD4, BioXCell, https://bioxcell.com/invivoplus-anti-mouse-cd4-bp0003-1;
2) InVivoPlus anti-mouse CD8α, BioXCell, https://bioxcell.com/invivoplus-anti-mouse-cd8a-bp0061;

The species and application of the following antibodies used for flow cytometry were validated by the manufacturer.
1) Anti-mouse CD11c, Biolegend, https://www.biolegend.com/en-us/products/fitc-anti-mouse-cd11c-antibody-1815;
2) Anti-mouse CD80, Biolegend, https://www.biolegend.com/en-us/products/pe-anti-mouse-cd80-antibody-43;
3) Anti-mouse CD86, Biolegend, https://www.biolegend.com/en-us/products/apc-anti-mouse-cd86-antibody-2896;
4) Anti-mouse CD11b, Biolegend, https://www.biolegend.com/en-us/products/pe-anti-mouse-human-cd11b-antibody-349;
5) Anti-mouse F4/80, Biolegend, https://www.biolegend.com/en-us/products/apc-anti-mouse-f4-80-antibody-4071;
6) Anti-mouse CD80, Biolegend, https://www.biolegend.com/en-us/products/percp-cyanine5-5-anti-mouse-cd80-antibody-4275;
7) Anti-mouse CD206, Biolegend, https://www.biolegend.com/en-us/products/percp-cyanine5-5-anti-mouse-cd206-mmr-antibody-8477;
8) Anti-mouse CD3, Biolegend, https://www.biolegend.com/en-us/products/fitc-anti-mouse-cd3-antibody-45;
9) Anti-mouse CD4, Biolegend, https://www.biolegend.com/en-us/products/percp-anti-mouse-cd4-antibody-4219;
10) Anti-mouse CD8a, Biolegend, https://www.biolegend.com/en-us/products/apc-anti-mouse-cd8a-antibody-150;
11) Anti-mouse Foxp3, Biolegend, https://www.biolegend.com/en-us/products/pe-anti-mouse-foxp3-antibody-4660;
12) Anti-mouse CD3, Biolegend, https://www.biolegend.com/en-us/products/percp-cyanine5-5-anti-mouse-cd3-antibody-5596;
13) Anti-mouse CD62L, Biolegend, https://www.biolegend.com/en-us/products/apc-cyanine7-anti-mouse-cd62l-antibody-3934;
14) Anti-mouse CD8a, Biolegend, https://www.biolegend.com/en-us/products/fitc-anti-mouse-cd8a-antibody-153;
15) Anti-mouse CD44, Biolegend, https://www.biolegend.com/en-us/products/pe-anti-mouse-human-cd44-antibody-2206;
16) Anti-mouse CD16/32, Biolegend, https://www.biolegend.com/en-us/products/purified-anti-mouse-cd16-32-antibody-190;
17) Anti-mouse SIINFEKL/H-2Kb, eBioscience, https://www.thermofisher.cn/cn/zh/antibody/product/OVA257-264-SIINFEKL-peptide-bound-to-H-2Kb-Antibody-clone-eBio25-D1-16-25-D1-16-Monoclonal/12-5743-81;

The species and application of the following antibodies used for immunohistochemistry assay were validated by the manufacturer.
1) Anti-mouse HSP70, Santa Cruz, https://www.scbt.com/p/hsp-70-hsc-70-antibody-w27?requestFrom=search;
2) Anti-mouse TNF-α, Abcam, https://www.abcam.cn/products/primary-antibodies/tnf-alpha-antibody-52b83-ab1793.html;

The species and application of the following antibodies used for immunofluorescence staining were validated by the manufacturer.
1) Anti-mouse F4/80, Abcam, https://www.abcam.cn/products/primary-antibodies/f480-antibody-ab100790.html;
2) Anti-mouse CD8 alpha, Abcam, https://www.abcam.cn/products/primary-antibodies/cd8-alpha-antibody-yts1694-ab22378.html;
3) Goat anti-rabbit IgG, H+L, Thermo Fisher Scientific, https://www.thermofisher.cn/cn/zh/antibody/product/Goat-anti-Rabbit-IgG-H-L-Highly-Cross-Adsorbed-Secondary-Antibody-Polyclonal/A32733;
4) Goat anti-rat IgG, H+L, Thermo Fisher Scientific, https://www.thermofisher.cn/cn/zh/antibody/product/Goat-anti-Rat-IgG-H-L-Secondary-Antibody-Polyclonal/A18866;

Eukaryotic cell lines

Policy information about cell lines and Sex and Gender in Research

Cell line source(s)	4T1, CT26, and HEK293 cells were supplied by the Chinese Academy of Sciences. 4T1 luciferase labeled cells (4T1-luc) were obtained from Shanghai Zhongqiao Xinzhou Biotechnology Co., Ltd. B16F10-OVA cells, a variant of the B16-F10 murine melanoma cell line that expresses OVA, were bought from Vigen Biotechnology (Zhenjiang) Co., Ltd and kindly gifted from Professor Cong Luo, Shenyang Pharmaceutical University.
Authentication	Cell line validation with short tandem repeat (STR) markers was conducted via Genetic Testing Biotechnology Corporation (Suzhou, China). In detail, eighteen STR loci were amplified using multiplex PCR. One additional marker (Human TH01) was used to screen for the presence of human species. The cell line sample was processed with ABI Prism 3130 XL Genetic Analyzer. Data were analyzed by Gene Mapper ID 3.2 software (Applied Biosystems). Appropriate positive and negative controls were run and confirmed for sample.
Mycoplasma contamination	All cell lines tested negative for mycoplasma contamination.
Commonly misidentified lines (See ICLAC register)	No commonly misidentified cells lines were used in the study.

Animals and other research organisms

Policy information about studies involving animals; ARRIVE guidelines recommended for reporting animal research, and Sex and Gender in Research

Laboratory animals	Female BALB/c and C57BL/6j mice (6-8 weeks old) were supplied by the Animal Center of Shenyang Pharmaceutical University (Shenyang, Liaoning, China). The living environment of animals were maintained at a temperature of ~25 ℃ and a humidity of 50 ± 5% with a 12 h light/dark cycle, with free access to standard food and water.
Wild animals	The study did not involve wild animals.
Reporting on sex	Although we have used single-sex animals in our research, we think that the research results were not only applicable to single-sex.
Field-collected samples	The study did not involve samples collected from the field.
Ethics oversight	All the animal experiments were conducted according to the Guidelines for the Care and Use of Laboratory Animals approved by the Institutional Animal Ethical Care Committee (IAEC) of Shenyang Pharmaceutical University.

Note that full information on the approval of the study protocol must also be provided in the manuscript.

Flow Cytometry

Plots

Confirm that:

- [x] The axis labels state the marker and fluorochrome used (e.g. CD4-FITC).
- [x] The axis scales are clearly visible. Include numbers along axes only for bottom left plot of group (a 'group' is an analysis of identical markers).
- [x] All plots are contour plots with outliers or pseudocolor plots.
- [x] A numerical value for number of cells or percentage (with statistics) is provided.

Methodology

Sample preparation	For tissue sample, the tissue was first mechanically disrupted from mice and divided into small pieces and homogenized in cold staining buffer to form single cell suspensions in the presence of digestive enzyme.
Instrument	BD FACSCalibur
Software	BD CellQuest Pro; Flowjo_V10
Cell population abundance	No sorting was performed.
Gating strategy	Generally, cells were first gated on FSC/SSC. Singlet cells were usually gated using FSC-H and FSC-A. Surface antigen gating was performed on the live cell population.

- [x] Tick this box to confirm that a figure exemplifying the gating strategy is provided in the Supplementary Information.
